# Simultaneous Identification of Functional Antigen-Specific CD8^+^ and CD4^+^ Cells after In Vitro Expansion Using Elongated Peptides

**DOI:** 10.3390/cells11213451

**Published:** 2022-10-31

**Authors:** Juliane Schuhmacher, Leon Kleemann, Jennifer Rebecca Richardson, Elisa Rusch, Hans-Georg Rammensee, Cécile Gouttefangeas

**Affiliations:** 1 Interfaculty Institute for Cell Biology, Department of Immunology, University of Tübingen, 72076 Tübingen, Germany; 2 Cluster of Excellence iFIT (EXC2180) “Image-Guided and Functionally Instructed Tumor Therapies”, University of Tübingen, 72076 Tübingen, Germany

**Keywords:** T cells, immunomonitoring, elongated peptides, ELISpot, intracellular cytokine staining

## Abstract

Elongated peptides (EPs), containing possibly one or multiple epitope/s, are increasingly used for the screening of antigen-specific CD8^+^ and CD4^+^ cell responses. Here, we present an in vitro protocol that allows the amplification of antigen-specific cells and the subsequent functional analysis of both T cell types using EPs. Known viral-derived epitopes were elongated to 20 mer EPs on the N-, C-, and both termini for HLA class I binders, or on the N- and C- termini for HLA class II binders. With EP stimulation only, the percentage of responding CD8^+^ T cells was dependent on the elongation site of the EP, whereas CD4^+^ T cell responses were completely lost in 22% of the tests performed ex vivo. A short-term amplification step plus the addition of a TLR3 agonist (Poly-ICLC) together with an increased EP concentration improved markedly the detection of CD8^+^ and CD4^+^ T cell reactivities.

## 1. Introduction

The identification of antigen-derived T cell epitopes, such as the mapping of virus- or tumor-derived epitopes and the monitoring of immunotherapy approaches, is essential to basic and clinical immunology. To assess functional antigen-specific T cells, methods such as the intracellular cytokine staining (ICS) assay or the interferon-(IFN-)γ Enzyme-Linked Immuno-Spot Assay (ELISpot) are widely used. Such tests can be performed ex vivo on whole blood or on isolated peripheral blood mononuclear cells (PBMCs). However, memory CD8^+^ and CD4^+^ T cells are generally present at low frequencies in the blood. To overcome the intrinsic detection limit of these read-out assays and increase the detection of rare T cells, an in vitro amplification is often performed prior to testing. This is done by stimulating the PBMCs with the antigen of choice at the beginning of the culture and by adding interleukin-(IL-)2. The culture is typically carried out for seven to twelve days before cell testing [1]. In our hands, the expansion rate of antigen-specific T cells over a 12-day stimulation period is donor- and antigen-dependent and varies between 10- and more than 1000-fold. Synthetic peptides represent the most accessible and convenient format for stimulating T cells in vitro. Since CD8^+^ and CD4^+^ T cells recognize short epitopes (classically 8–12 amino acids (aa) on HLA class I and 13–18 aa on HLA class II), “minimal” synthetic short peptides can be used when the epitopes and/or the HLA restriction are known or should be precisely identified. This is, for example, the case for the in vitro monitoring of anti-cancer vaccines containing a mixture of HLA class I and class II binding peptides [2,3,4]. When the exact peptide is not known, pools of (overlapping) elongated peptides (EPs; >15 aa) can be used. EPs potentially contain both HLA class I and class II epitopes and can be used for assessing CD8^+^ and CD4^+^ T cell specificities in vitro [5]. They represent a suitable format to limit the number of tests to be performed and are therefore often used in large-scale screening studies, e.g., for assessing the immunogenicity of pathogen-derived proteins [6,7,8,9,10]. They are also used in experimental vaccination approaches for the stimulation of both T cell types in vivo [6,11,12,13,14,15,16]. EPs of 15–20 aa are considered to be of optimal length for recognition by CD4^+^ T cells. However, in most cases, they stimulate CD8^+^ T cells less effectively than their short epitope counterpart, although this can be, at least in part, compensated by an increased peptide concentration [8,9,17,18]. The simultaneous identification of both CD8^+^ and CD4^+^ antigen-specific T cells using a single peptide format is therefore imperfect. Hence, one method is to use EPs for the monitoring of antigen-specific CD4^+^ T cells, together with predicted short peptides or overlapping short peptide pools for the identification of CD8^+^ T cell responses [11,19]. A protocol that would allow the optimized and simultaneous identification of antigen-specific CD8^+^ and CD4^+^ T cells using a single EP format is of high interest for the immune monitoring field.

Singh et al. established a protocol for the ex vivo detection of low-frequency antigen-specific CD8^+^ and CD4^+^ T cells using overlapping EPs of 30 aa. Autologous adherent monocytes were stimulated with GM-CSF, peptide-pulsed, and used as antigen-presenting cells (APCs) to stimulate the non-adherent PBMC fraction. A higher peptide concentration (50 µg/mL) and the addition of Poly-IC, a Toll-like receptor (TLR) 3 agonist, together with Roferon-A (IFN-α), led to an improvement in the detection of CD8^+^ T cell reactivities, in the range of 50–90% of the responses detected when using the matched short peptides [20].

Here, we present an optimized protocol for the amplification of T cells in bulk PBMCs (no monocyte isolation is needed) during 12 days and their testing by ICS and/or ELISpot using EPs. First, we compared CD8^+^ and CD4^+^ T cell responses against known viral-derived HLA class I and class II epitopic peptides and their elongated 20 aa long versions (N-, C-, N- and C-terminal extensions). We show that not only CD8^+^ T cell responses but also CD4^+^ T cell responses can be suboptimally detected with EPs. The addition of Poly-ICLC on day 1 of the culture and an increase in the EP peptide concentration for the pre-stimulation and the read-out were sufficient to detect CD8^+^ and CD4^+^ responses at adequate levels. This optimized protocol should be especially suitable when the number of PBMCs is limited, or when the number of peptides to be tested is substantial, i.e., for large-scale epitope discovery or for the monitoring of patient-derived samples after vaccination with EP pools.

## 2. Materials and Methods

### 2.1. PBMC Isolation, Cell Freezing, and Thawing

Buffy coats, mononuclear blood cell concentrates, or leukaphereses from healthy volunteers were obtained from the Center for Clinical Transfusion Medicine Tübingen. Participants gave informed consent and the study was approved by the ethics committee of the Medical Faculty at the University of Tübingen, projects 713/2018BO2 and 156/2012B01. PBMCs from *n* = 10 healthy donors were isolated within 1 day after blood drawing. The blood products were diluted 1:4 (buffy coats, leukaphereses) or 1:8 (mononuclear blood cell concentrates) with PBS (in-house preparation from 10x, Lonza, Walkersville, MD, USA), and PBMCs were isolated using density centrifugation (Biocoll, Merck, Darmstadt, Germany). PBMCs were washed twice with PBS and up to 30 × 10^6^ cells were resuspended in 1 mL freezing medium (heat-inactivated: h.i.) fetal bovine serum (FBS, Capricorn Scientific, Ebsdorfergrund, Germany) containing 10% dimethyl sulfoxide (DMSO, Sigma-Aldrich, St. Louis, MO, USA). Cryovials (NuncTM, Sigma-Aldrich) were placed into a cryo container (Nalgene^®^ Mr. Frosty, Sigma-Aldrich), stored for up to four days at −80 °C, and transferred to liquid nitrogen (−196 °C). PBMCs were pre-screened for the presence of virus-specific T cells by ICS (stimulation with the short peptidic epitopes) and multimer stainings (for CD4^+^ and CD8^+^ T cells, respectively). For all experiments, cells were thawed using IMDM (Gibco BRL, Invitrogen, Grand Island, NY, USA) supplemented with 2.5% h.i. human male AB serum (Sigma-Aldrich), 100 U/mL Penicillin/0.1 mg/mL Streptomycin (Pen/Strep; Sigma-Aldrich), 50 µm β-Mercaptoethanol (β-ME; Merck), and 3 µg/mL DNAse I (Sigma-Aldrich).

### 2.2. Synthetic Peptides

Known short HLA class I (HLA-A*0201, *n* = 3) and HLA class II (HLA-DR, *n* = 3) T cell epitopes were synthesized in-house using an automated peptide synthesis system (ABI 433A, Applied Biosystems, Foster City, USA). Purity was assessed by reverse-phase liquid chromatography (e296, Waters). Each of these peptides was elongated to a 20 mer EP according to the respective protein sequence, at the N-, C-, and N- and C-termini for HLA class I (+11 aa in total), or at the N- and C-termini for HLA class II peptides (+5 to 6 aa in total). All EPs were ordered from JPT Peptide Technologies (Berlin, Germany). Lyophilized peptides were diluted in sterile MilliQ H_2_O containing 10% DMSO. Peptide source, sequence, aa position, elongation site, code, and purity are shown in Table 1.

### 2.3. Twelve-Day In Vitro Stimulation of PBMCs

For the in vitro amplification of virus-specific cells, 1.0–3.5 × 10^6^ or 3.6–6.5 × 10^6^ PBMCs/well were seeded in T cell medium (TCM; IMDM supplemented with 100 U/mL Pen/0.1 mg/mL Strep, 50 µM β-ME, and 10% h.i. human AB serum) in a 48- or 24-well plate, respectively (Cellstar^®^, Greiner Bio-One, Frickenhausen, Germany) and cultured overnight at 37 °C, 7.5% CO_2_. No pre-adherent step was performed. On day 1, untouched PBMCs were stimulated with 1, 5, 10, or 50 µg/mL peptides, as indicated in the figure legends. Additionally, cells were supplemented with the single addition of Poly-ICLC (P; Poly-IC stabilized with Poly-L-lysine and carboxymethyl cellulose [21], Hiltonol^®^; 20 µg/mL; Oncovir, Washington, DC, USA), with GM-CSF (G; 0.8 U/µL; PeproTech, Rocky Hill, NJ, USA), with the combination of both (P+G, either single or multiple additions, same concentration as for the single addition), or were left in media alone (standard condition). On days 3, 5, 7, and 9, 2 ng/mL recombinant human IL-2 (R&D Systems) was added to the culture. Cells were split 1:2 on day 5, 7, or 9 if the cell layer was >70% confluent. This protocol successfully expands both CD4^+^ and CD8^+^ memory T cells [1,2,22,23,24]. On day 12, cells were harvested, counted manually using a Neubauer chamber and 0.1% trypan blue (Sigma Aldrich), and further analyzed for their functionality either with ICS or ELISpot. For the ELISpot analysis, the in vitro culture was performed in two independent replicates for each condition.

### 2.4. Intracellular Cytokine Staining Assay for T Cell Detection

PBMCs were either thawed approximately 8 h before the ex vivo ICS, resuspended in TCM containing 1 µg/mL DNase I and rested at 37 °C, 7.5% CO_2_ until use, or directly tested on day 12 after the in vitro culture. Here, 0.5–2 × 10^6^ cells/well were seeded in a 96-well round-bottom plate (Cellstar^®^, Greiner Bio-One) and stimulated with either 10 µg/mL short peptides or with 10 or 50 µg/mL EPs, as indicated in the figure legends. DMSO (10% in H_2_O) and Staphylococcus enterotoxin B (SEB; 10 µg/mL Sigma-Aldrich) were used as negative and positive controls, respectively. The protein transport inhibitors Brefeldin A (10 µg/mL, Sigma-Aldrich) and Golgi Stop (BD, Heidelberg, Germany) were added, and cells were incubated for 12 h at 37 °C, 7.5% CO_2_. For the staining, cells were washed in FACS buffer (PBS without Ca/Mg (Lonza), 0.02% NaN_3_ (Roth), 2 mM EDTA (Sigma-Aldrich), and 2% h.i. FBS) and stained extracellularly for 20 min at 4 °C. After one washing step, cells were fixed and permeabilized (Cytofix/Cytoperm, BD) for 20 min at 4 °C, washed once with permeabilization buffer (PBS 1x, 0.02% NaN_3_, 0.5% BSA, and 0.1% saponin (Sigma-Aldrich), and stained intracellularly for 20 min at 4 °C. All antibodies (Table 2) were pre-titrated. The cells were acquired on a LSRFortessa^TM^ SORP (BD) using the DIVA software (Version 6.1.3). The data analysis was performed with the FlowJo software (Version 10.6.1) and responding cells were defined as TNF^+^IFN-γ ^+^. An exemplary gating strategy is shown in Figure S1A,B for the ex vivo ICS. For the ICS analysis after the 12-day in vitro culture, the lymphocyte (FSC-A/SSC-A) gate was enlarged. All dot plots were manually audited.

### 2.5. Flow Cytometry Staining of Immune Cell Subsets

After the 12-day in vitro cultivation, 0.5 × 10^6^ cells per test were used for the staining of immune cell subsets. Fc receptors were blocked by adding 10 µL Fc block (BD, final concentration 0.25 mg/mL) for 10 min at RT. Without a washing step, cells were subsequently stained with pre-titrated antibodies (Table S1) for 20 min at 4 °C, for T cell, B cell, NK cell, monocyte, and dendritic cell identification. A representative gating strategy and the results are shown in Figure S2A. Cells were washed with FACS buffer and acquired on a LSRFortessaTM SORP (BD) equipped with DIVA (Version 6.1.3). The data analysis was performed with the FlowJo software (Version 10.6.1), and all dot plots were manually audited.

### 2.6. Enzyme-Linked Immunospot Assay

Our IFN-γ ELISpot protocol is described in detail elsewhere [2,22]. The ELISpot was performed in technical duplicates. Here, 50,000 cells/well were seeded for the virus-derived peptide stimulation and 300,000 cells/well for the background assessment (10% DMSO, negative control). Phytohemagglutinin-L (PHA, 10 µg/mL, Sigma-Aldrich) was used as a positive control. Spots were counted with the ImmunoSpot series 6 ultra-V analyzer (CTL Europe GmbH) according to the laboratory standard protocol. No cut-off was set for the spot count.

### 2.7. Statistics

The GraphPad Prism 6 (version 6.01) software was used for statistical analysis. Normal distribution was checked by the Kolmogorov–Smirnov test with Dallal–Wilkinson– Lilliefors correction. For comparison of two parameters, a Mann–Whitney test was used, and for single-parametric multi-comparison, a one-way ANOVA was performed. For multiple comparison of matched measurements, the Friedman test was used. Statistical differences were considered significant for *p* ≤ 0.05 (*) or *p* < 0.01 (**) or *p* < 0.001 (***). Information on the statistical test and number of data points tested is provided in the legend for each experiment.

## 3. Results

### 3.1. Ex Vivo Detection of Viral-Specific T Cells Using EPs Alone Can Be Suboptimal

To assess whether CD8^+^ and CD4^+^ T cell reactivities can readily be detected when using EPs instead of short epitopes, we used known HLA class I and class II viral-derived epitopes from the cytomegalovirus (CMV), Epstein–Barr virus (EBV), and Influenza virus (INF) as model antigens. Short epitopic peptides were elongated to 20 aa EPs according to the source protein sequences either at the N-, C-, or both N- and C-termini for HLA class I peptides, or at the N- and C-termini for HLA class II peptides (Table 1). As a first step, we assessed the ex vivo immune responses (TNF^+^IFN-γ ^+^ cytokine response in ICS) of CD8^+^ or CD4^+^ cells against the short peptides and the respective EPs in PBMCs of healthy donors (HD; *n* = 3 EPs or *n* = 1 EP per specificity for elongated HLA class I or class II peptides, respectively; each tested in *n* = 3 HDs). All PBMCs had been pre-tested for virus-specific cells (frequencies ranged from 0.02 to 8.80% peptide-specific cells within the CD8^+^ and/or CD4^+^ cell subsets). EPs were used at two purity grades (>90% and >50%). A representative gating strategy and examples of the flow cytometry results are shown in Figure S1A,B.

**Figure 1:**
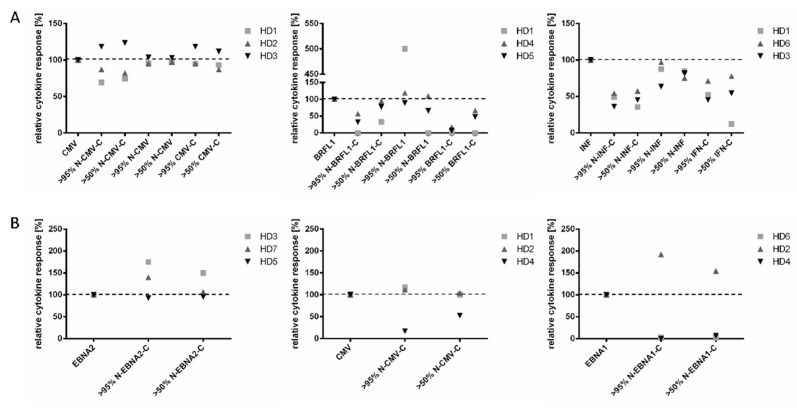
Ex vivo detection of virus-specific T cell responses using EPs can be suboptimal. PBMCs from healthy donors (*n* = 7 HDs in total, *n* = 3 per specificity) were stimulated with 10 µg/mL HLA class I and class II viral-derived epitopes from CMV, EBV (BRFL1/EBNA1/EBNA2), and Influenza virus (INF) of short length, or with their respective EP version (20 mers, N-peptide source-C: N- and C-terminal elongated; N-peptide source: N-terminal elongated; peptide source-C: C-terminal elongated) for 12 h. Two EP purities (>50% and >90%) were used. The percentages of peptide-specific (background cytokine production in the negative control was subtracted) CD8^+^ (**A**) and CD4^+^ (**B**) cells that produced both cytokines (IFN-γ^+^TNF^+^) were calculated. The anti-EP cytokine response is depicted related to the response to the matched short peptide (range 0.012–2.22% of IFN^+^TNF^+^ cells within the CD4^+^ or CD8^+^ subsets), which is normalized to 100%. The full gating strategy is depicted in Figure S1A,B and frequencies of specific cells are given in Table S2.

Antigen-specific CD8^+^ cells (Figure 1A) and CD4^+^ cells (Figure 1B; see Figure S1C,D for all reactivities pooled together) could be detected after stimulation with the short peptides (set to 100%) in almost all cases (except HD1 response against BRFL1). We also detected a response against most EPs. However, the frequencies of reacting T cells were often reduced compared to that seen against the corresponding short peptides and, occasionally, even completely lost (exemplary frequencies of peptide-specific CD4^+^ T cells: Figure 1B right panel: HD4/HD6, peptide EBNA1: 0.090%/0.013%, N-EBNA1-C 0.00%/0.00%). The loss of response against the EBV-derived EP N-EBNA1-C in two of three donors was especially unexpected, since elongation was only three amino acids at the N- and C-termini. We further observed that HLA class I epitopes elongated in the N-terminal mostly induced cytokine levels comparable to that of the matched short peptide, whereas the presence of a C-extension led to decreased recognition of nearly 60% in mean for two out of the three peptides tested (BRFL1 and INF, but not CMV; Figure 1A and Figure S1C). There was no statistically significant difference in the cytokine levels induced by the EPs of the two purities (paired Student’s *t*-test; Figure S1E); therefore, only the peptides with the highest purity were used for all following experiments. These results clearly show that the ex vivo monitoring of CD8^+^ or CD4^+^ T cells with elongated (i.e., 20 mer) peptides repeatedly misses T cell responses and needs further improvement.

### 3.2. The Addition of Poly-ICLC and GM-CSF Improves the Detection of Viral-Specific T Cells with EPs after 12 Days of In Vitro Expansion

**Figure 2:**
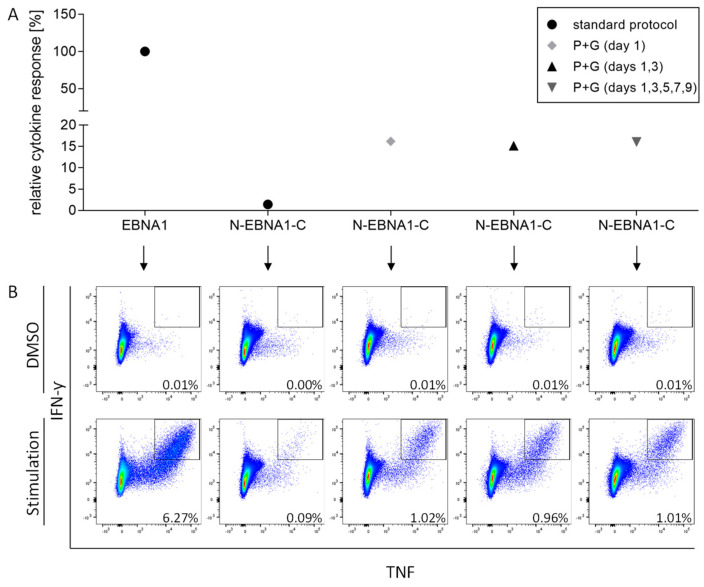
The addition of Poly-ICLC and GM-CSF can improve the detection of viral-specific T cells after in vitro expansion using EPs. PBMCs from healthy donor 4 (HD4) were stimulated with the short HLA class II peptide from EBV EBNA1 (EBNA1) or its N+C-terminal EP version (N-EBNA1-C), both at a concentration of 5 µg/mL on day 1 without or with the addition of 20 µg Poly-lCLC and 0.8 U/µL GM-CSF (P+G). P+G was added once or repeatedly during the 12-day culture. On day 12, cells were re-stimulated in ICS with the same peptide as on day 1 (10 µg/mL short peptide; 50 µg/mL EP). Single cells, live CD4^+^ lymphocytes, were analyzed for their cytokine production (IFN-γ^+^TNF^+^ cells). (**A**) Specific cytokine response (percentage of IFN-γ^+^TNF^+^CD4^+^ cells after subtracting the background cytokine production in the negative control (DMSO)) after EP stimulation without (standard protocol, black dot) or with the addition of P+G on day 1 (light grey diamond), days 1 and 3 (black triangle), or days 1, 3, 5, 7, and 9 (inverted dark grey triangle) relative to the cytokine response to the matched short peptide set to 100% (standard protocol, black dot). (**B**) The respective flow cytometry dot plots are shown for each condition, either for the negative control (DMSO; upper panel), or after peptide stimulation (lower panel). Percentages indicate the frequencies of IFN-γ^+^TNF^+^ events within the CD4^+^ cell population. Full gating strategy is depicted in Figure S1A,B.

In order to overcome the detection limit of functional assays, many laboratories, including our own, use a short amplification step (synthetic peptides + IL-2) to increase the frequency of antigen-specific T cells within PBMCs [1,2,22,23,25]. It was shown that a high peptide concentration loaded onto monocytes, together with the addition of IFN-α, GM-CSF, and a TLR3 ligand, is favorable for the detection of effector T cells, particularly CD8^+^ cells [20]. We therefore tested whether the addition of GM-CSF and Poly-ICLC in our PBMC culture could improve the expansion and subsequent detection of antigen-specific T cells. We used cells from donor HD4, who had shown a response against the short HLA class II EBNA1 peptide but no response against the elongated N-EBNA1-C EP in the ex vivo testing (Figure 1B, right panel). HD4 PBMCs were stimulated with 5 µg/mL of the EBNA1 or N-EBNA1-C peptides on day 1 without further addition (our standard setting), or in the presence of Poly-ICLC and GM-CSF (P+G). P+G were either added once on day 1 or on several days of the in vitro culture (days 1, 3, 5, 7, and 9). At day 12, cells were re-stimulated with the same peptide as on day 1 and analyzed by ICS. For the re-stimulation, we used our standard concentration of 10 µg/mL for the short peptide, whereas we increased the peptide concentration of the EP to 50 µg/mL [20]. CD4^+^ T cells stimulated with the EP alone produced cytokines; however, the response was approximately 70-fold lower as compared to that obtained against the matched short peptide (approximately 6.3% to 0.09% of the CD4^+^ T cells; Figure 2). With the addition of P+G, we observed an 11-fold (approximately 1% of the CD4^+^ cells) increase in cytokine-producing cells after EP stimulation compared to the condition without P+G. No difference in the frequency of cytokine-producing cells was observed between the single or multiple additions of P+G. Although this T cell response was still approximately six-fold lower as compared to that against the short peptide, cells were now readily detectable (Figure 2B). Hence, the addition of P+G can improve the detection of antigen-specific cells stimulated with EPs.

### 3.3. The Single Addition of Poly-ICLC Is Superior to the Combination of Poly-ICLC and GM-CSF for T Cell Amplification with EPs

**Figure 3:**
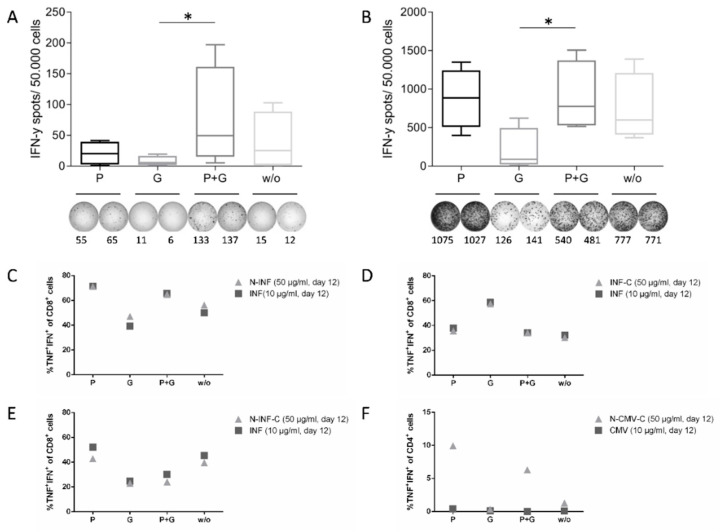
The single addition of Poly-ICLC is superior to the combination of Poly-ICLC and GM-CSF for the detection of viral-specific CD4^+^ and CD8^+^ T cells using EPs. PBMCs were stimulated with virus-derived HLA class I and HLA class II EPs (10 µg/mL of 20 mers; N- and/or -C-terminal elongations are indicated) on day 1. In addition to the peptide stimulation on day 1, Poly-ICLC (20 µg/mL, P), GM-CSF (0.8 µg/mL, G), or the combination of P+G (same concentration as for the single addition) were added. Control stimulation was performed with peptide only (*w*/*o*). (**A**,**B**) IFN-γ ELISpot analysis at day 12 of the background spot counts ((**A**), negative control; 300,000 cells/well seeded, normalized to 50,000 cells) or spot counts after restimulation with 50 µg/mL EPs ((**B**), 50,000 cells/well seeded). EPs were CMV (N-CMV-C) and EBV EBNA2 (N-EBNA2-C) (HLA class II peptides), or CMV (N-CMV) and Influenza virus (N-INF) (HLA class I peptides). *n* = 2 HDs. Tests were performed in duplicate. Box and whisker plots of the mean IFN-γ spot counts per 50,000 cells are shown (min to max and median values are indicated). Statistical analysis: Friedman test with post-test Dunn’s multiple comparison. * *p* ≤ 0.05. Representative ELISpot wells and spot counts (technical replicates) of PBMCs from HD5 for the negative control or the EP N-INF stimulation are shown with spot counts. (**C**–**F**) ICS results. Cells were re-stimulated on day 12 with 10 µg/mL short peptide or 50 µg/mL of the respective EP. Specific frequencies (background subtracted) of cytokine^+^ (IFNγ^+^TNF^+^) CD8^+^ (HD1; (**C**–**E**)) or CD4^+^ (HD4, (**F**)) cells are shown relative to the responses against the matched short peptides. Absolute frequencies with short peptide stimulation: 27.7% IFNγ^+^TNF^+^CD8^+^ (HLA class I, INF); 4.70% IFNγ^+^TNF^+^CD4^+^ (HLA class II, CMV). Full gating strategy is depicted in Figure S1A,B.

To identify the minimum essential compound(s) that needed to be added to the T cell amplification step, we stimulated PBMCs with different peptides either alone, in combination with P+G, or with the single compounds P or G. IFN-γ ELISpot and ICS were performed on day 12 to identify functional antigen-specific cells. For the ELISpot, we seeded 300,000 cells/well for the unstimulated control, as this is a cell number that we usually plate, e.g., for the monitoring of cancer peptide vaccines (Figure 3A, spot counts normalized to 50,000 cells). For the cells stimulated with peptides, we seeded 50,000 cells/well to reach a spot count within the quantification range of the ELISpot reader (Figure 3B). PBMCs of *n* = 2 HDs (HD5, HD8) were stimulated, each with one HLA class I (N-INF (HD5), N-CMV (HD8)) and one HLA class II (N-EBNA2-C (HD5), N-CMV-C (HD8)) EP on day 1 and day 12 of the in vitro culture. The highest background (unstimulated cells) spot counts were observed for the combination of P+G (median 49.6), followed by the condition without any addition (median 25.3), and the single addition of Poly-ICLC (median 20.1). The single addition of GM-CSF demonstrated the lowest background spot numbers (median 5.6) (Figure 3A). The specific spot counts for the stimulated cells were the highest for the condition with Poly-ICLC (median 861.3), followed by the conditions with P + G (median 727.1), without addition (median 573.9), and with GM-CSF alone (median 84.0) (Figure 3B).

For the ICS, we stimulated cells from *n* = 2 HDs with HLA class I EP variants (N-INF, INF-C, N-INF-C (Figure 3C–E)) or one HLA class II EP variant (CMV, N-CMV-C (Figure 3F)) on day 1. On day 12, cells were re-stimulated with the same EPs as on day 1 or with the respective short peptide version as a control. In three out of four tests, the response was the highest after expansion with EP plus Poly-ICLC, as shown in Figure 3C–F (HLA class I and class II peptides). Similar results were obtained with two further donors tested with EBV-derived peptides (Figure S3).

Considering all tests (Figure S4A,B show all data points together), the highest frequencies were detected in five of eight tests with the single addition of Poly-ICLC, followed by the condition without any addition (2/8), and that with the single addition of GM-CSF (1/8). The addition of P+G led, in no case, to the best frequency of activated T cells. The background cytokine production was overall low, with no marked difference between conditions (data not shown). Taking together the results obtained with the two read-out methods, the single addition of Poly-ICLC was superior to the other conditions.

### 3.4. Optimal Peptide Concentration Finding for Simultaneous Read-Out of CD8^+^ and CD4^+^ T Cells with EPs

**Figure 4:**
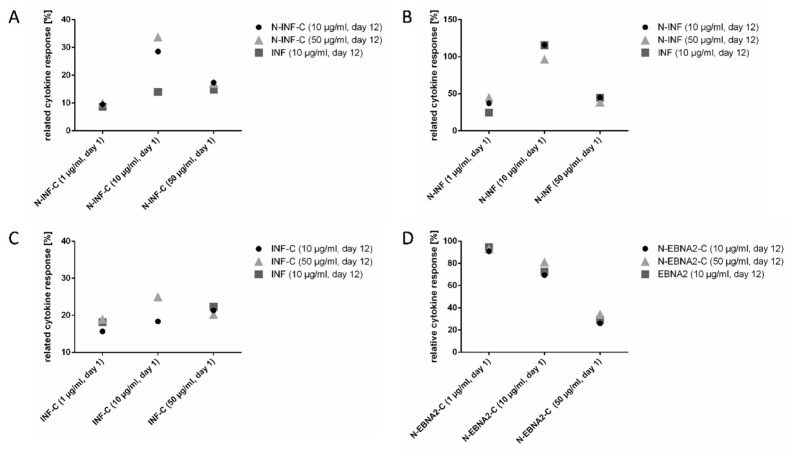
Identification of the optimal EP concentration for the read-out of viral-specific CD8^+^ and CD4^+^ cells after in vitro expansion. PBMCs were cultured with INF- or EBV-derived short peptides or matched EPs (20 mers, N- and/or -C-terminal elongations are indicated) for 12 days. On day 1, cells were stimulated with 1 µg/mL (HLA class I) or 5 µg/mL (HLA class II) short peptide (standard concentrations) or with 1, 10, or 50 µg/mL of the EPs plus 20 µg/mL Poly-ICLC (*x*-axis). On day 12, cells were re-stimulated in the ICS with 10 µg/mL short peptide or with 10 µg/mL or 50 µg/mL of the respective EP (graph legends). Single live CD8^+^ or CD4^+^ lymphocytes were analyzed for their specific IFN-γ and TNF expression (percentage of IFN-γ^+^TNF^+^ cells; background of the negative control was subtracted). Shown are the percentages of double-positive cytokine CD8^+^ cells ((**A**–**C**), HD9) or CD4^+^ cells ((**D**), HD7) relative to the cytokine response detected with the standard protocol. Absolute frequencies with short peptide stimulation: 11.9% IFN-γ^+^TNF^+^ CD8^+^ (INF); 16.3% IFN-γ^+^TNF^+^ CD4^+^ (EBV EBNA2). Full gating strategy is depicted in Figure S1A,B.

The final step was to determine the optimal EP concentration that should be used together with Poly-ICLC for the simultaneous detection of cytokine-producing CD8^+^ and CD4^+^ cells. For this, we stimulated PBMCs from *n* = 4 HDs with EPs from *n* = 3 viral sources (EBV, CMV, and INF, two for each HD) resulting in altogether *n* = 5 test conditions for HLA class I peptides and *n* = 2 test conditions for HLA class II peptides. We tested different EP concentrations on day 1 and day 12 (1, 10, 50 µg/mL), which were in the range of peptide concentrations usually applied for activating T cells (17, 19, 21). In all cases, Poly-ICLC was added on day 1. Matched short peptides were used as controls for the T cell stimulation on day 1 and for the read-out on day 12 (1 µg/mL and 10 µg/mL, respectively). Representative results are shown in Figure 4A–C for the stimulation of CD8^+^ cells with *n* = 3 INF-derived EP variants and in Figure 4D for the stimulation of CD4^+^ cells with *n* = 1 HLA class II EBV-derived EP. Pooled data of all donor/peptide combinations are presented in Figure S4C,D. The results obtained for the HLA class I N-CMV and CMV-C EPs, as well as for the HLA class II EBV N-EBNA-2-C peptide, are shown in Figure S5. In all cases, T cell responses against the EPs could be detected. Taking all experiments for HLA class I EPs into account, the highest responses against the EPs were detected when cells were stimulated with 10 µg/mL EP on day 1 and 50 µg/mL on day 12 (three out of five tests). For the HLA class II CMV-derived peptide, the highest response against the EP was detected for 1 µg/mL (day 1) and 10 or 50 µg/mL on day 12 (two out of two tests). Overall, the CD8^+^ and CD4^+^ T cell responses against EPs reached between 25% and 204% of that obtained against the short epitopes. Although the optimal conditions might differ for CD8^+^ and CD4^+^ cells, 10 µg/mL EP on day 1 and 50 µg/mL EP on day 12 appear to be the best condition for the detection of T cell responses in both subsets simultaneously.

## 4. Discussion

The identification and quantification of antigen-specific T cells are essential for T-cell-based immunotherapies and epitope mapping studies. In many instances, T cells of interest are rare, and their robust and sensitive in vitro measurement remains a technical challenge. Therefore, approaches are being adopted to increase the frequency of these rare cells, such as the in vitro amplification with synthetic peptides before cells are assessed.

Here, we present an optimized protocol for the pre-sensitization and functional read-out of antigen-specific CD8^+^ and CD4^+^ cells together using elongated peptides. As model antigens, we used described HLA class I and class II virus-derived short epitopes that were elongated to 20 aa at the N-, C-, or N+C-termini and used for stimulating PBMCs from pre-selected healthy donors with various CD4^+^ and/or CD8^+^ T cell antigen specificities. Frequencies of responding T cells were assessed by multiparametric ICS and by IFN-γ ELISpot. First, we considered whether EPs are suitable for assessing CD8^+^ and CD4^+^ responses in an ex vivo setting. For most specificities, effector cells could indeed be detected when using either the short epitopic peptides or the respective EPs. However, there were notable exceptions, both for HLA class I and for HLA class II binders. For all but one donor/HLA class I epitope combination, N-terminus elongated peptides showed comparable results to the short peptide versions, whereas the response was decreased by up to 93% if epitopes were elongated on the C-terminus (C or N+C EPs; Figure S1C). Our results are in accordance with previous observations showing that the capacity of recalling CD8^+^ T cell responses depends on the elongation site (N, C, N+C) [26]. However, it is not yet fully clear whether the processing of EPs for presentation on the HLA class is proteasome-dependent, or if long peptides are trimmed by proteases, either extracellularly or in endosomal or lysosomal compartments [5,26]. To test proteasome dependency in our setting, we incubated PBMCs with the proteasome inhibitor Epoxomycin (5 µM) 30 min prior to EP addition (*n* = 1 EP (CMV-C); *n* = 2 HD). No effect on the activation of antigen-specific CD8^+^ cells was observed (data not shown). This suggests proteasome-independent peptide processing, possibly occurring through extracellular peptidases. For HLA class II epitopes, it is well described that HLA class II molecules can accommodate ligands of 8–25 residues. Examples of this “length permissiveness” have been repeatedly revealed by ligandomics studies, which commonly identify several length variants of the same core sequence as HLA class II binding peptides [27,28]. Hence, the drastic loss of CD4^+^ T cell reactivities when using EPs for activation was unanticipated.

We next aimed at establishing a single protocol for the expansion and read-out of both HLA class I and class II restricted T cell responses simultaneously based on the following considerations: (1) EPs are efficiently processed and cross-presented by human monocyte-derived DCs for CD8 T cell presentation [5], and (2) an increased EP concentration and the addition of GM-CSF, IFN-α, and Poly-IC to monocytes used as APCs improved the detection of T cell responses ex vivo [20]. We therefore tested whether the addition of GM-CSF, which is routinely used to differentiate monocytes into DCs in vitro, and Poly-ICLC, a toll-like receptor ligand serving as an adjuvant in recent peptide vaccine studies, could improve the expansion of specific T cells when using EPs instead of exact short epitopes. Although the cytokine response for the single addition of Poly-ICLC was, in median, comparable to our standard condition (*w*/*o*) for the read-out of CD8^+^ T cells in ICS, the addition of Poly-ICLC alone was found to be the best condition for the read-out of CD8^+^ and CD4^+^ T cells together. Notably, no change in the frequencies of total CD8^+^ and CD4^+^ cells was observed after the in vitro stimulation period across the different conditions (Figure S6). This suggests that Poly-ICLC, which has been shown to activate an innate immune response in vivo [29], may also improve the expansion of antigen-specific T cells in vitro. To further dissect the effect of Poly-ICLC in vitro, we also analyzed the immune cell subset composition after T cell amplification (Figure S2 and Table S1). We observed a significant 2.4-times increase in the frequency of B cells (from median 0.56% to 1.34%), together with enhanced median fluorescence intensity of HLA-DR on these cells. The percentages of the other APC subsets tested, i.e., monocytes and DCs, were not affected. This suggests that EPs could be presented by B cells in our setting, as has been previously shown by others using activated B cells [5,30]. Since blood human B cells generally do not express TLR3 [31] and do not respond to Poly-IC by proliferation or differentiation [32], they are most likely not stimulated directly by Poly-ICLC. Hence, the indirect activation of B cells, possibly by other TLR3-expressing cells within the PBMCs (T cells, myeloid DCs, or NK cells [33]), may take place during the 12-day preculture step.

We show that EPs can be efficiently used both for in vitro CD8^+^ and CD4^+^ T cell amplification and at the read-out step. However, EPs do not allow the identification of the exact epitopes recognized by T cells. We therefore tested whether it is possible to use the EPs for the amplification step (day 1) and the short counterpart for the read-out test on day 12. For HLA class I restricted peptides, this was possible in all cases, whereas reactivities against the corresponding short HLA class II peptides were occasionally lost. This might indicate that several final epitopes could be processed and presented from the EP sequence and recognized differently in individual donors expressing different HLA class II allelic products [34].

**Figure 5:**
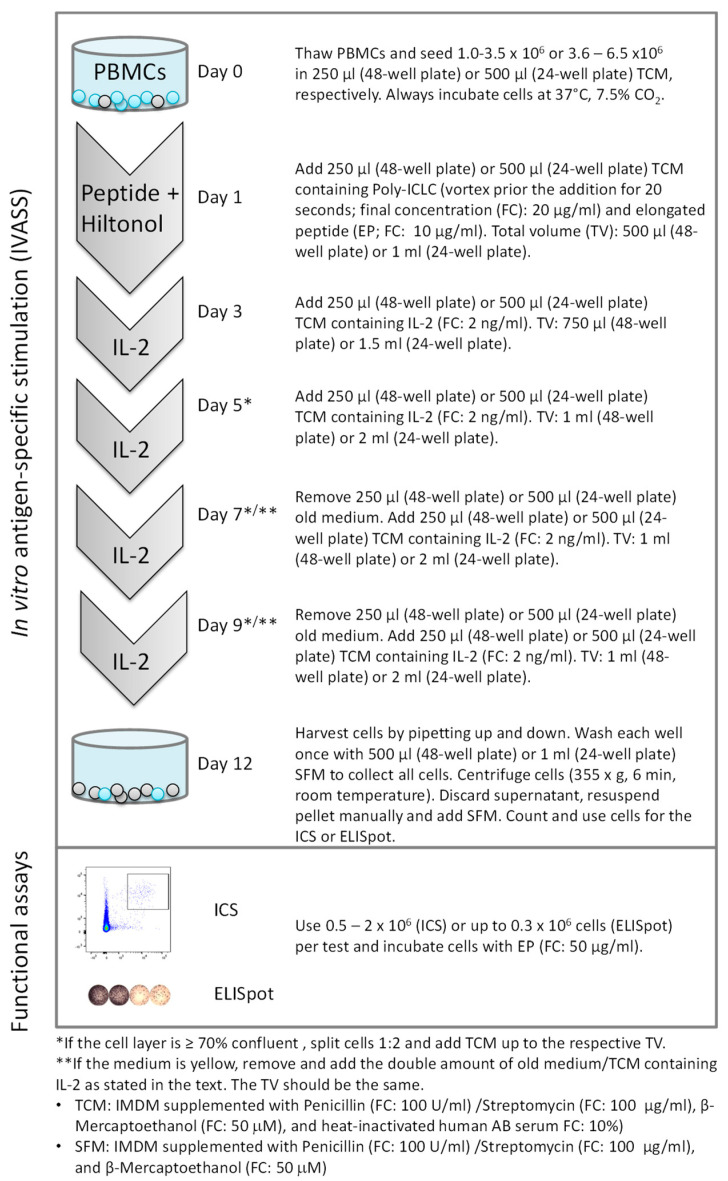
Protocol scheme for the simultaneous detection of antigen-specific CD8^+^ and CD4^+^ cells after in vitro expansion using elongated peptides.

Based on our results, we propose the use of 10 µg/mL EP plus 20 µg/mL Poly-ICLC on day 1, and 50 µg/mL EP on day 12, for the simultaneous screening of antigen-specific CD8^+^ and CD4^+^ T cell responses (a protocol scheme is shown in Figure 5). All peptides included in our study represent well-described immunodominant epitopes from virus proteins that strongly bind to HLA-A*0201 (for the three CD8^+^ T cell epitopes used) and very likely also with high affinity to HLA class II allelic products (for the three CD4^+^ T cell epitopes). We have already applied the same conditions for simultaneous CD8^+^ and CD4^+^ T cell assessment (10 µg/mL EP on day 1 and 50 µg/mL EP to read-out) to monitor tumor antigen-specific T cells in cancer patients. In a recent study, prostate carcinoma patients were vaccinated with a single 20 aa EP (derived from the RhoC tumor antigen) emulsified in Montanide. The in vitro monitoring demonstrated that 86% of the patients did respond to the vaccine by developing anti-vaccine CD4^+^ T cells, and one simultaneous CD8^+^ cell response was also detected [35]. With the same protocol, we also verified that PBMC T cells from a patient vaccinated with a survivin-derived class II epitope (15 mer) could be detected with the cognate EP (20aa). The frequencies of specific CD4^+^ cells were similar using the short and the long peptides (data not shown). Hence, the new, improved protocol is not only suitable for the read-out of viral-specific responses but also for that of anti-cancer responses.

## 5. Conclusions

We have established an improved assay for the simultaneous detection of low-frequency antigen-specific CD8^+^ and CD4^+^ T cells in ICS and ELISpot using elongated peptides. This protocol should be useful for both large-scale epitope discovery for, e.g., pathogens, and for the immune monitoring of T-cell-based immunotherapies, especially anti-cancer vaccines.

## Figures and Tables

**Table 1:** Virus-derived synthetic peptides used in the study.

Restriction	Source (Virus/Protein)	Peptide Length (aa)	Sequence *	Peptide Position (aa) in Source Protein	Elongation Site	Code	Purity>50%	Purity>95%
HLA class I (HLA-A*0201)	CMV pp65 strain AD169 Uniprot P06725	9	**NLVPMVATV**	495–503		CMV	-	>95
		20	GILAR**NLVPMVATV**QGQNLK	490–509	N + C	N-CMV-C	89	98
		20	WPPWQAGILAR**NLVPMVATV**	484–503	N	N-CMV	70	96
		20	**NLVPMVATV**QGQNLKYQEFF	503–514	C	CMV-C	67	98
	Matrix Influenza strain A/Puerto Rico/8/1934 H1N1 Uniprot P03485	9	**GILGFVFTL**	58–66	-	INF	-	100
		20	SPLTK**GILGFVFTL**TVPSER	53–72	N + C	N-INF-C	78	90
		20	KTRPILSPLTK**GILGFVFTL**	47–66	N	N-INF	66	98
		20	**GILGFVFTL**TVPSERGLQRR	53–77	C	INF-C	63	98
	EBV BRFL1 strain B95-8 Uniport P03209	9	**YVLDHLIVV**	109–117	-	BRFL1	86	-
		20	PIVMR**YVLDHLIVV**TDRFFI	104–123	N + C	N-BRFL1-C	53	98
		20	ACSIACPIVMR**YVLDHLIVV**	98–117	N	N-BRFL1	83	95
		20	**YVLDHLIVV**TDRFFIQAPSN	109–128	C	BRFL1-C	52	96
HLA class II	CMV pp65 strain AD169 Uniprot P06725-1	15	**YQEFFWDANDIYRIF**	510–524	-	CMV	-	95
		20	NLK**YQEFFWDANDIYRIF**AE	507–526	N + C	N-CMV-C	74	98
	EBV EBNA2 strain B95-8 Uniprot P12978	15	PRSPTVFYNIPPMPL	276–290	-	EBNA2	81	-
		20	SPE**PRSPTVFYNIPPMPL**PP	273–292	N + C	N-EBNA2-C	60	99
	EBV EBNA1 strain B95-8 Uniprot P03211	14	**KTSLYNLRRGTALA**	514–527	-	EBNA1	72	-
		20	GGS**KTSLYNLRRGTALA**IPQ	511–530	N + C	N-EBNA1-C	89	96

CMV: cytomegalovirus; EBV: Epstein–Barr virus; * for epitope references, see www.iedb.org (accessed on 7 September 2022). aa in bold show described epitopes.

**Table 2:** Monoclonal antibody panel for ICS.

Staining Step	Marker	Fluorochrome	Clone	Manufacturer	Cat. No.
Extracellular staining	Live/Dead	Zombie Aqua	-	BioLegend	423102
	CD4	APC-Cy7	RPA-T4	BD	557871
	CD8	BV605	RPA-T8	BioLegend	301040
Intracellular staining	TNF	Pacific Blue	MAB11	BioLegend	502920
	IFN-γ	FITC	B27	BD	554700

## Data Availability

Raw data and flow cytometry plots are available upon request.

## Supplementary Materials

The following supporting information can be downloaded at: https://www.mdpi.com/article/10.3390/cells11213451/s1, Figure S1: Gating strategy, exemplary dot plots, and pooled results for the ex vivo ICS with comparison of the relative cytokine response after stimulation using two EP purities; Figure S2: Immune cell populations detected with or without addition of Poly-ICLC during T cell expansion; Figure S3: The single addition of Poly-ICLC is superior to the combination of Poly-ICLC and GM-CSF for the detection of viral-specific CD4^+^ and CD8^+^ T cells using EPs; Figure S4: The addition of Poly-ICLC and an increased EP concentration allow the simultaneous amplification of antigen-specific CD8+ and CD4+ T cells and their detection in ICS; Figure S5: Identification of the optimal EP concentration for the read-out of functional virus-specific CD8^+^ and CD4^+^ cells after in vitro expansion; Figure S6: The single addition of Poly-ICLC during T cell expansion does not alter the CD8^+^ (A) and CD4^+^ (B) cell frequencies; Table S1: Monoclonal antibody panel for the detection of immune cell subsets; Table S2: Absolute percentages of IFN-γ^+^TNF^+^ CD8^+^ and CD4^+^ cells after short peptide or EP stimulation detected in ex vivo ICS.
