# Supplementary material for: Simultaneous Identification of Functional Antigen-Specific CD8+ and CD4+ Cells after In Vitro Expansion Using Elongated Peptides
Source: Cells. 2022 Oct 31;11(21):3451. doi: 10.3390/cells11213451 (PMC9654851; doi:10.3390/cells11213451)
Supplement: Supplementary file 1 [file cells-11-03451-s001.zip › cells-1933277-SM.pdf]

cells

MDPI

Supplementary Material

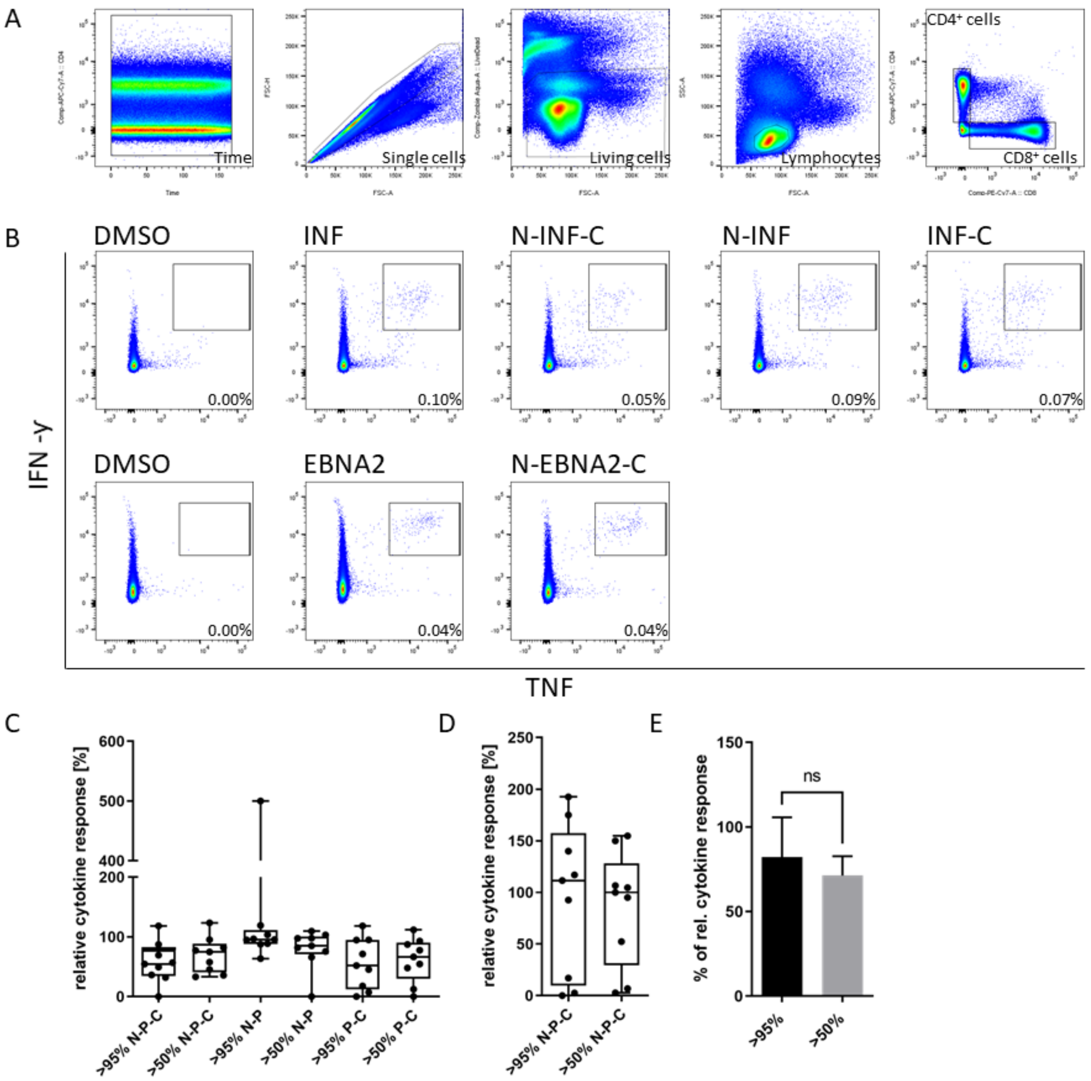

Figure S1. Gating strategy, exemplary dot plots and pooled results for the ex vivo ICS with comparison of the relative cytokine response after stimulation using two EP purities. (**A**) Gating strategy from left to right: time, single cells, living cells, and lymphocytes, which were further gated for their CD8 or CD4 coreceptor expression. (**B**) Exemplary results for the IFN-γ and TNF coexpression in $CD8^+$ cells (upper row; Healthy donor 6 (HD6)) and CD4+ cells (lower row, HD5) after stimulation with DMSO (10% in water; negative control) or viral-derived peptides from the Influenza (INF) and EBV (EBNA2) viruses of short and elongated length (20 mers, N-peptide source-C: N- and C-terminal elongated; N- peptide source: N-terminal elongated; peptide source-C: C-terminal elongated). Percentages indicate IFN-γ^+ TNF^+ cells within the $CD4^+$/$CD8^+$ cell populations. (**C**/**D**) Box and whisker plots show pooled data from Figure 1A for $CD8^+$ T cells (**C**) and from Figure 1B for $CD4^+$ T cells (**D**); min to max values and lines indicate medians. (**E**) Median with 95% CI of the percentage of cytokine response after EP stimulation related to the short peptide, which is normalized to 100%. Two EP purities, >50 and ≥90%, were used. n = 36 donor/peptide (HLA-class I and -class II) combinations per purity. Statistical test: Mann-Whitney test, ns = not significant.

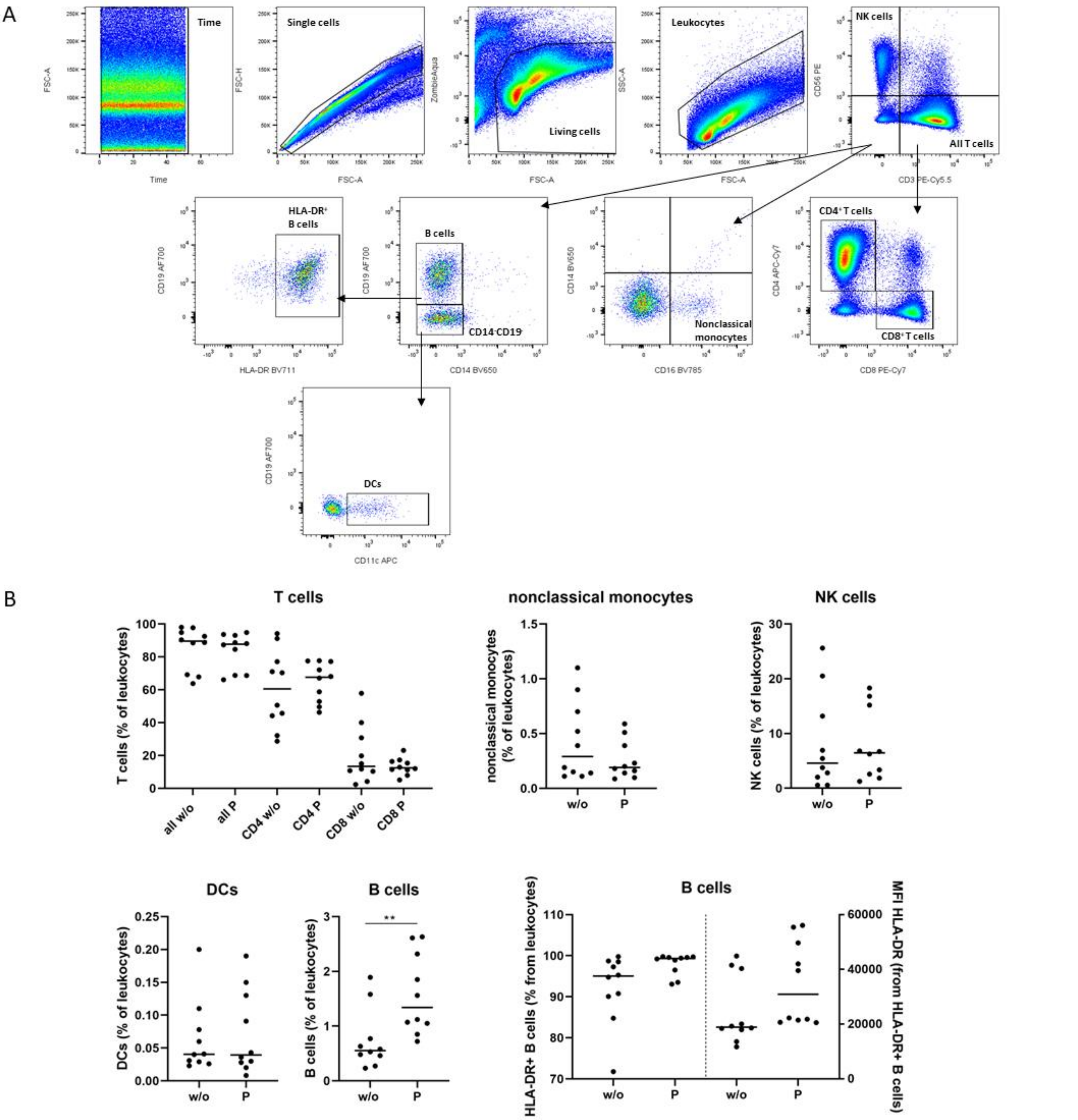

Figure S2. Immune cell populations detected with or without addition of Poly-ICLC during T-cell expansion. PBMCs from 4 HDs were stimulated with virus-derived peptides and with (P) or without (w/o) 20 µg Poly-ICLC added on day 1 of the in vitro culture. On day 12, cells were harvested and 500.000 cells were stained to identify immune cell subsets. (**A**) Gating strategy from left to right: time, single cells, living cells, and leukocytes. Leukocytes were further gated for NK cells (CD3$^-$CD56$^+$) and T cells (CD3$^+$CD56$^-$). T cells were subsequently gated for CD4$^+$ T cells (CD3$^+$CD56$^-$CD4$^+$) and CD8$^+$ T cells (CD3$^+$CD56$^-$CD8$^+$). CD56$^-$ and CD3$^-$ cells were gated for non-classical monocytes (CD3$^-$CD56$^-$CD14$^-$CD16$^{+)}$ and B cells (CD3$^-$CD56$^-$CD14$^-$CD19$^+$). CD14$^-$ and CD19$^-$ cells were analyzed for DCs (CD3$^-$CD56$^-$CD19$^-$CD14$^-$CD11c$^+$). (**B**) Scatter-plot graphs show the frequency of the various immune cell subsets. (**C**) HLA-DR expression within B cells (left *Y*-axis) and corresponding HLA-DR median fluorescence (MFI) (right *Y*-axis). Each point represents one test condition and bars indicate medians. Statistical analysis: Mann-Whitney test. ** p <0.01.

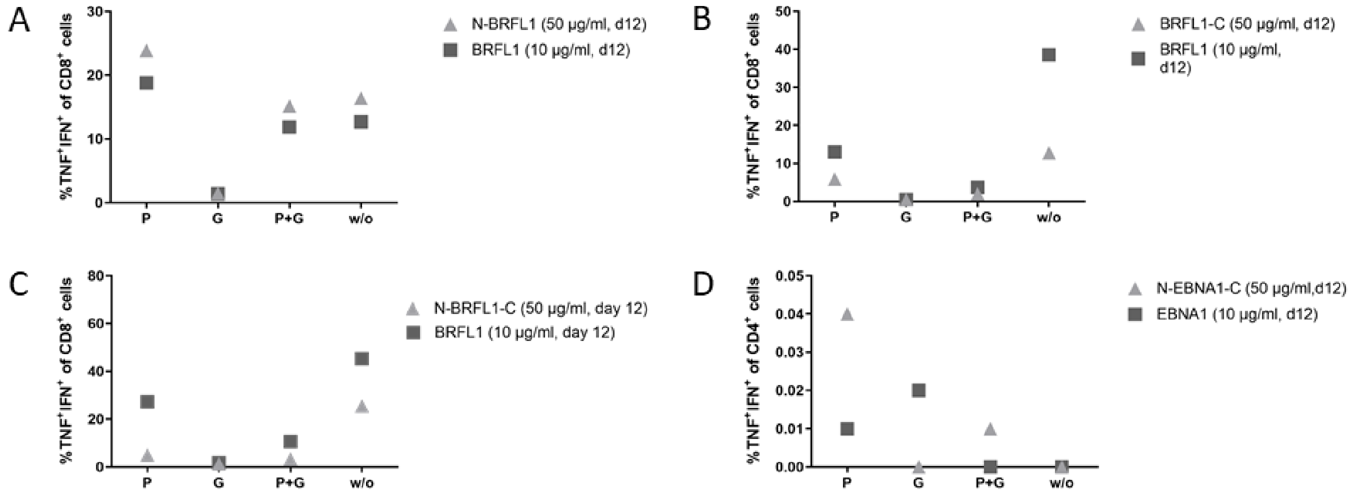

Figure S3. The single addition of Poly-ICLC is superior to the combination of Poly-ICLC and GM-CSF for the detection of viral-specific CD4$^+$ and CD8$^+$ T cells using EPs. PBMCs were stimulated with virus-derived HLA-class I and HLA-class II EPs (10 µg/mL of the 20 mers, N- and/or -C-terminal elongations are indicated) on day 1. In addition to the peptide stimulation on day 1, Poly-ICLC (20 µg/mL, P), GM-CSF (0.8 µg/mL, G), or the combination of P + G (same concentration as for the single addition) were added. Control stimulation was performed with peptide only (w/o). (**A**–**D**) ICS results. Cells were re-stimulated on day 12 with 10 µg/mL short peptide or 50 µg/mL of the respective EP. Specific frequencies (background subtracted) of cytokine (IFNγ$^+$TNF$^+$) CD8$^+$ (HD5; **A**–**C**) or CD4$^+$ (HD6, **D**) cells are shown relative to the responses again the matched short peptides. Absolute frequencies with short peptide stimulation: 16,2% IFNγ$^+$TNF$^+$CD8$^+$ (INF); 0.85% IFNγ$^+$TNF$^+$CD4$^+$ (CMV). Full gating strategy is depicted in Figure S1A,B.

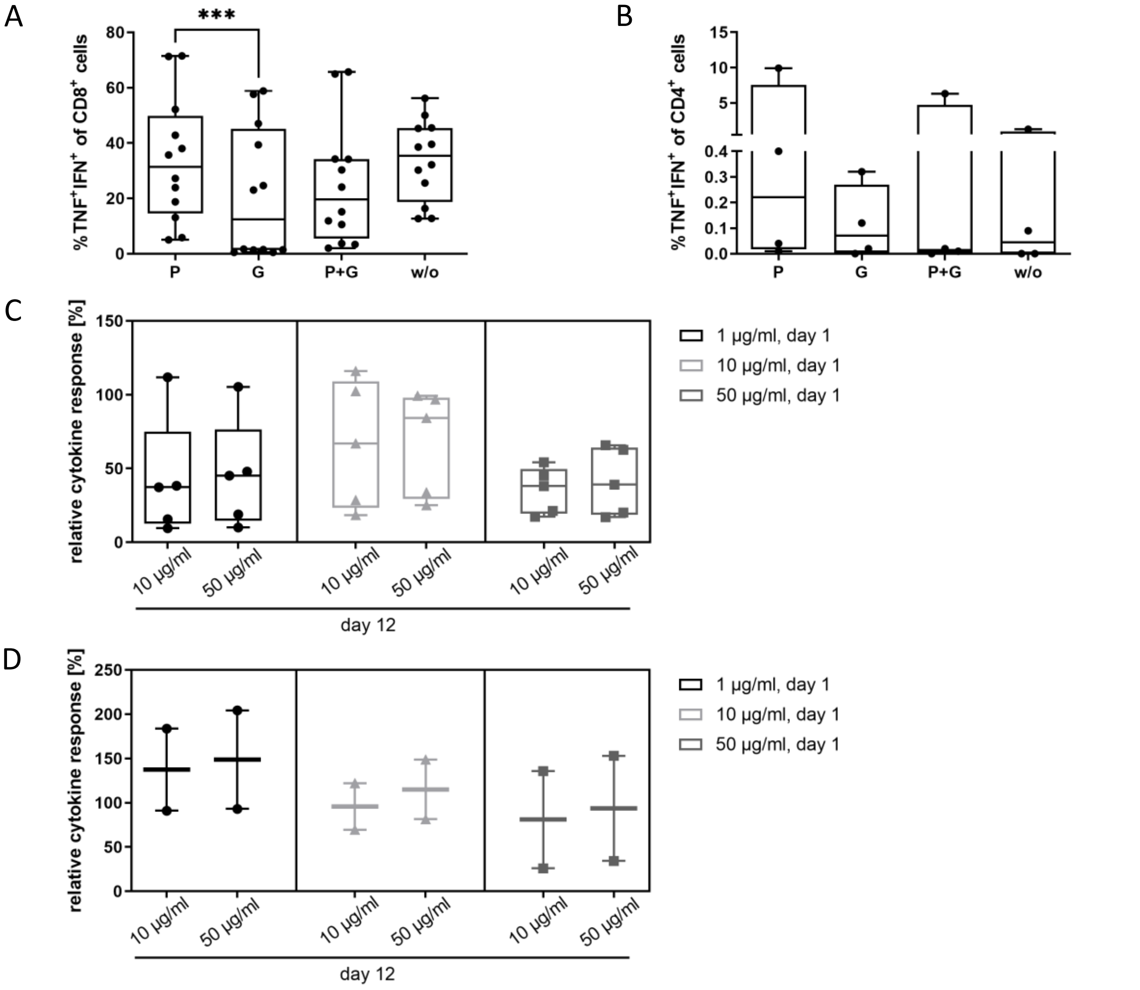

Figure S4. The addition of Poly-ICLC and an increased EP concentration allows the simultaneous amplification of antigen-specific CD8+ and CD4+ T cells and their detection in ICS. (**A**/**B**) Box and whisker plots show the pooled data from Figure 3C–E and Figure S3A–C for CD8+ T cells (**A**) and Figure 3F and Figure S3D for CD4+ T cells (**B**). Stimulation conditions: EPs + Poly-ICLC (P), GM-CSF (G), the combination of Poly-ICLC and GM-CSF (P + G) or without addition (w/o). Lines indicate medians. Statistics: Friedman test. *** $p < 0.001$ (**C**/**D**) Min to Max plots showing the pooled data for the stimulation of the cells with EPs on day 1 and day 12 of the in vitro culture of Figure 4A–C and Figure S5A/B for CD8+ T cells (**C**) and Figure 4D and Figure S5C for CD4+ T cells (**D**). Min to max values and lines indicate the medians.

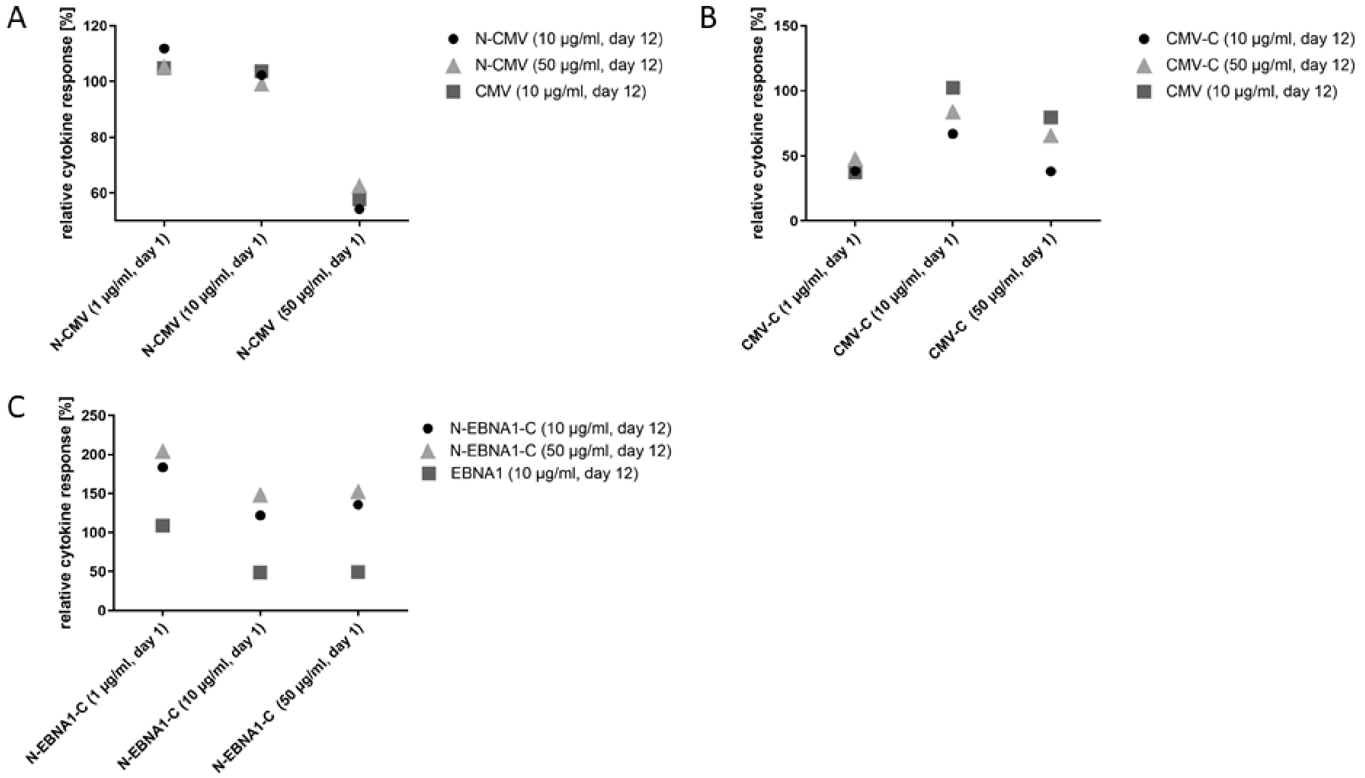

Figure S5. Identification of the optimal EP concentration for the read-out of functional virus-specific CD8⁺ and CD4⁺ cells after in vitro expansion. PBMCs were cultured with CMV- or EBV-derived short peptides or matched EPs (20 mers, N- and/or -C-terminal elongations are indicated) for 12 days. On day 1, cells were stimulated with 1 µg/mL (HLA-class I) or 5 µg/mL (HLA-class II) short peptides (standard concentrations) or with 1, 10, or 50 µg/mL of the EP plus the addition of 20 µg/mL Poly-ICLC (*x*-axis). On day 12, cells were re-stimulated with 10 µg/mL of the short peptide or with 10 or 50 µg/mL of the respective EP before ICS analysis. Single live CD8⁺ or CD4⁺ lymphocytes were analyzed for their specific IFN-γ and TNF expression (percentage of IFN-γ⁺TNF⁺ cells; background of the negative control was subtracted). The percentages of double positive cytokine CD8⁺ cells (**A**/**B**, donor (HD2) or CD4⁺ cells (**C**, HD10) relative to the cytokine response detected by the standard protocol (cells stimulated with the short peptide on day 1 (1/5 µg/mL) and on day 12 (10 µg/mL)) are shown. Absolute frequencies with short peptide stimulation: 58.7% IFN-γ⁺TNF⁺ CD8⁺ (CMV); 6.4% IFN-γ⁺TNF⁺ CD4⁺ (EBV EBNA2).

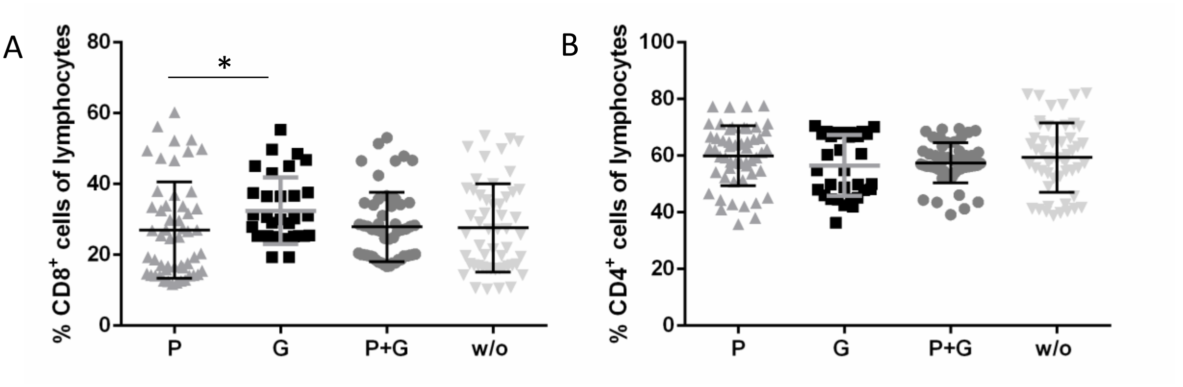

Figure S6. The single addition of Poly-ICLC during T cell expansion does not alter the CD8⁺ (A) and CD4⁺ (B) cell frequencies. On day 1 of the 12-day in vitro culture, PBMCs were stimulated with virus-derived peptides of short length or with their respective EP version (HLA-class I restricted epitopes derived from EBV BRFL1 and Influenza virus: N- and C-terminal, N-terminal, and C-terminal elongated; HLA-class II restricted epitopes derived from EBV EBNA1 and CMV: N- and C-terminal). In addition to the peptide stimulation, Poly-ICLC (20 µg/mL, P), GM-CSF (0.8 µg/mL, G),

or the combination of P + G (same concentration as for the single addition) or no addition (w/o) was tested. On day 12, cells were re-stimulated with 10 µg/mL of the short peptide, 50 µg/mL of the respective elongated version, or left unstimulated (10% DMSO), then ICS was performed. Frequencies of $CD4^+$ and $CD8^+$ cells within living lymphocytes are shown. $n = 7$ donors (except GM-CSF alone: $n = 4$ donors), $n = 2$ tests per donor and condition (short peptide) and $n = 3$ tests per donor and condition (EP). Statistical analysis: Kruskal-Wallis test with Dunn's multiple comparison post-test. * $p \leq 0.05$.

Table S1. Monoclonal antibody panel for the detection of immune cell subsets.

	Marker	Fluorochrome	Clone	Manufacturer	Cat. No.
Extracellular staining	Live/Dead	Zombie Aqua	-	BioLegend, San Diego, CA, USA	423102
	CD3	PE-Cy5.5	SK7	eBioscience, San Diego, CA, USA	35-0036-42
	CD4	APC-Cy7	RPA-T4	BD, Heidelberg, Germany	557871
	CD8	PE-Cy7	SFCI21Thy2D3	BeckmanCoulter, Brea, CA. USA	737661
	CD11c	APC	3.9	BioLegend	301613
	CD14	BV650	M5E2	BioLegend	301835
	CD16	BV786	B73.1	BioLegend	360734
	CD19	AF700	HIB19	BD	561031
	CD56	PE	B159	BD	555516
	HLA-DR	BV711	G46-6	BD	563696

Table S2. Absolute percentages of IFN-γ^+TNF$^+$ $CD8^+$ and $CD4^+$ cells after short peptide or EP stimulation detected in ex vivo ICS (Figure 1).

Peptide Source (HLA-Class Restriction)	Healthy Donor (HD)	Pepitde (Sequence)	Peptide Purity	Absolute IFN-γTNF Double Positive Cytokine Response (Background Subtracted)*
CMV (HLA-class I)	HD1	CMV (NLVPMVATV)	>95%	2.29%
		N-CMV-C (GILARNLVPMVATVQGQNLK)	>90%	1.59%
		N-CMV-C (GILARNLVPMVATVQGQNLK)	>50%	1.71%
		N-CMV (WPPWQAGILARNLVPMVATV)	>90%	2.19%
		N-CMV (WPPWQAGILARNLVPMVATV)	>50%	2.24%
		CMV-C (NLVPMVATVQGQNLKYQEFF)	>90%	2.17%
		CMV-C (NLVPMVATVQGQNLKYQEFF)	>50%	2.13%
	HD2	CMV (NLVPMVATV)	>95%	0.40%
		N-CMV-C (GILARNLVPMVATVQGQNLK)	>90%	0.35%
		N-CMV-C (GILARNLVPMVATVQGQNLK)	>50%	0.33%
		N-CMV (WPPWQAGILARNLVPMVATV)	>90%	0.38%
		N-CMV (WPPWQAGILARNLVPMVATV)	>50%	0.39%
		CMV-C (NLVPMVATVQGQNLKYQEFF)	>90%	0.38%
		CMV-C (NLVPMVATVQGQNLKYQEFF)	>50%	0.35%
	HD3	CMV (NLVPMVATV)	>95%	2.22%
		N-CMV-C (GILARNLVPMVATVQGQNLK)	>90%	2.63%
		N-CMV-C (GILARNLVPMVATVQGQNLK)	>50%	2.74%
		N-CMV (WPPWQAGILARNLVPMVATV)	>90%	2.30%
		N-CMV (WPPWQAGILARNLVPMVATV)	>50%	2.29%

		CMV-C (NLVPMVATVQGQNLKYQEFF)	>90%	2.63%
		CMV-C (NLVPMVATVQGQNLKYQEFF)	>50%	2.48%
EBV (HLA-class I)	HD1	BRFL1 (YVLDHLIVV)	86%	0.06%
		N-BRFL1-C (PIVMRYVLDHLIVVTDRFFI)	>90%	0.00%
		N-BRFL1-C (PIVMRYVLDHLIVVTDRFFI)	>50%	0.02%
		N-BRFL1 (ACSIACPIVMRYVLDHLIVV)	>90%	0.30%
		N-BRFL1 (ACSIACPIVMRYVLDHLIVV)	>50%	0.00%
		BRFL1-C (YVLDHLIVVTDRFFIQAPSN)	>90%	0.00%
		BRFL1-C (YVLDHLIVVTDRFFIQAPSN)	>50%	0.00%
	HD4	BRFL1 (YVLDHLIVV)	86%	0.21%
		N-BRFL1-C (PIVMRYVLDHLIVVTDRFFI)	>90%	0.12%
		N-BRFL1-C (PIVMRYVLDHLIVVTDRFFI)	>50%	0.20%
		N-BRFL1 (ACSIACPIVMRYVLDHLIVV)	>90%	0.25%
		N-BRFL1 (ACSIACPIVMRYVLDHLIVV)	>50%	0.23%
		BRFL1-C (YVLDHLIVVTDRFFIQAPSN)	>90%	0.04%
		BRFL1-C (YVLDHLIVVTDRFFIQAPSN)	>50%	0.14%
	HD5	BRFL1 (YVLDHLIVV)	86%	0.18%
		N-BRFL1-C (PIVMRYVLDHLIVVTDRFFI)	>90%	0.06%
		N-BRFL1-C (PIVMRYVLDHLIVVTDRFFI)	>50%	0.14%
		N-BRFL1 (ACSIACPIVMRYVLDHLIVV)	>90%	0.16%
		N-BRFL1 (ACSIACPIVMRYVLDHLIVV)	>50%	0.12%
		BRFL1-C (YVLDHLIVVTDRFFIQAPSN)	>90%	0.01%
		BRFL1-C (YVLDHLIVVTDRFFIQAPSN)	>50%	0.09%
INF (HLA-class I)	HD1	INF (GILGFVFTL)	100%	0.73%
		N-INF-C (SPLTKGILGFVFTLTVPSER)	>90%	0.36%
		N-INF-C (SPLTKGILGFVFTLTVPSER)	>50%	0.26%
		N-INF (KTRPILSPLTKGILGFVFTL)	>90%	0.64%
		N-INF (KTRPILSPLTKGILGFVFTL)	>50%	0.62%
		INF-C (GILGFVFTLTVPSERGLQRR)	>90%	0.38%
		INF-C (GILGFVFTLTVPSERGLQRR)	>50%	0.09%
	HD6	INF (GILGFVFTL)	100%	0.09%
		N-INF-C (SPLTKGILGFVFTLTVPSER)	>90%	0.05%
		N-INF-C (SPLTKGILGFVFTLTVPSER)	>50%	0.05%
		N-INF (KTRPILSPLTKGILGFVFTL)	>90%	0.09%
		N-INF (KTRPILSPLTKGILGFVFTL)	>50%	0.07%
		INF-C (GILGFVFTLTVPSERGLQRR)	>90%	0.07%
		INF-C (GILGFVFTLTVPSERGLQRR)	>50%	0.07%
	HD3	INF (GILGFVFTL)	100%	0.11%
		N-INF-C (SPLTKGILGFVFTLTVPSER)	>90%	0.04%
		N-INF-C (SPLTKGILGFVFTLTVPSER)	>50%	0.05%
		N-INF (KTRPILSPLTKGILGFVFTL)	>90%	0.07%
		N-INF (KTRPILSPLTKGILGFVFTL)	>50%	0.09%
		INF-C (GILGFVFTLTVPSERGLQRR)	>90%	0.05%
		INF-C (GILGFVFTLTVPSERGLQRR)	>50%	0.06%
EBV EBNA2 (HLA-class II)	HD3	EBNA2 (PRSPTVFYNIPPMPL)	81%	0.01%
		N-EBNA2-C (SPEPRSPTVFYNIPPMPLPP)	>90%	0.02%
		N-EBNA2-C (SPEPRSPTVFYNIPPMPLPP)	>50%	0.02%
	HD7	EBNA2 (PRSPTVFYNIPPMPL)	81%	0.02%
		N-EBNA2-C (SPEPRSPTVFYNIPPMPLPP)	>90%	0.02%

		N-EBNA2-C (SPEPRSPTVFYNIPPMPLPP)	>50%	0.02%
	HD5	EBNA2 (PRSPTVFYNIPPMPL)	81%	0.04%
		N-EBNA2-C (SPEPRSPTVFYNIPPMPLPP)	>90%	0.04%
		N-EBNA2-C (SPEPRSPTVFYNIPPMPLPP)	>50%	0.04%
CMV (HLA-class II)	HD1	CMV (YQEFFWDANDIYRIF)	95%	0.18%
		N-CMV-C (NLKYQEFFWDANDIYRIFAE)	>90%	0.21%
		N-CMV-C (NLKYQEFFWDANDIYRIFAE)	>50%	0.18%
	HD2	CMV (YQEFFWDANDIYRIF)	95%	0.09%
		N-CMV-C (NLKYQEFFWDANDIYRIFAE)	>90%	0.10%
		N-CMV-C (NLKYQEFFWDANDIYRIFAE)	>50%	0.09%
	HD4	CMV (YQEFFWDANDIYRIF)	95%	0.07%
		N-CMV-C (NLKYQEFFWDANDIYRIFAE)	>90%	0.01%
		N-CMV-C (NLKYQEFFWDANDIYRIFAE)	>50%	0.03%
EBV EBNA1 (HLA-class II)	HD6	EBNA1 (KTSLYNLRRGTALA)	72%	0.01%
		N-EBNA1-C (GGSKTSLYNLRRGTALAIPQ)	>90%	0.00%
		N-EBNA1-C (GGSKTSLYNLRRGTALAIPQ)	>50%	0.00%
	HD2	EBNA1 (KTSLYNLRRGTALA)	72%	0.01%
		N-EBNA1-C (GGSKTSLYNLRRGTALAIPQ)	>90%	0.01%
		N-EBNA1-C (GGSKTSLYNLRRGTALAIPQ)	>50%	0.01%
	HD4	EBNA1 (KTSLYNLRRGTALA)	72%	0.09%
		N-EBNA1-C (GGSKTSLYNLRRGTALAIPQ)	>90%	0.00%
		N-EBNA1-C (GGSKTSLYNLRRGTALAIPQ)	>50%	0.01%

* background cytokine production in the negative control was subtracted.
